# Qualitative and quantitative assessment of infraoccluded deciduous teeth: a systematic review

**DOI:** 10.1186/s13005-024-00469-3

**Published:** 2024-10-30

**Authors:** Teresa Temming, Susanne Waldmann, Anahita Jablonski-Momeni, Heike Korbmacher-Steiner

**Affiliations:** 1 Department of Orthodontics, Clinic of Dentistry, Philipps University Marburg, Georg-Voigt-Str. 3, 35039 Marburg, Germany; 2 Central Medical Library, University Library of Marburg, Philipps University Marburg, Conradistr. 3a, 35032 Marburg, Germany

**Keywords:** Tooth ankylosis, Deciduous teeth, Infraocclusion, Alveolar bone growth, Systematic review

## Abstract

**Background:**

Infraocclusion of deciduous teeth is a frequent dental anomaly caused by ankylosis accompanied by local growth disturbance. During puberal growth spurt an increasing progression of infraocclusion is detected. The clinical classification of ankylosed deciduous teeth varies considerably among scientific studies. The aim of this paper is to present an up-to-date overview of the variety of methods.

**Methods:**

The systematic literature search followed the PRISMA guidelines and included the analysis of the following databases and study registries: PubMed (MEDLINE), the Cochrane Library, Web of Science, Embase.com and ClinicalTrials.gov from database inception until September 23, 2024. Studies that investigated at least one ankylosed deciduous tooth per participant in a quantitative or qualitative manner were included. Studies that evaluated only histological data were excluded. Controlled and uncontrolled clinical studies, retrospective studies, observational studies and cross-sectional studies were included. The studies were restricted to English and German languages. Case reports, case series, comments, expert opinions, letters to the editor, literature reviews and studies enrolling less than 10 patients or 10 infraoccluded teeth in total were excluded.

**Results:**

Of 5645 records, 42 papers qualified for the final analysis. The evaluation of infraoccluded deciduous teeth was mainly (*n* = 37) performed by quantitative and semiquantitative assessment of the extent of infraposition at the occlusal level. The measurement reference differed considerably. Fewer studies have analyzed ankylosed deciduous teeth at the alveolar level by examining the contour of the alveolar ridge (*n* = 7) or the height of the alveolar process (*n* = 5). Even fewer studies (*n* = 4) have performed qualitative analysis at the skeletal level by evaluating the influence of the vertical skeletal growth pattern on the incidence of ankylosed deciduous teeth.

**Conclusions:**

To carry out a comprehensive evaluation of infraoccluded deciduous teeth, an assessment of the occlusal, alveolar and potentially skeletal levels is advisable. Radiographic investigations i.e. panoramic radiographs are therefore essential as a supplement to clinical examination. There is a need for standardization and objectification of the methods for the classification of infraoccluded deciduous teeth to give a general recommendation of clinical performance.

**Registration:**

This systematic review has been registered in the international prospective register of systematic reviews (PROSPERO) under the registration number: CRD42024555842.

**Supplementary Information:**

The online version contains supplementary material available at 10.1186/s13005-024-00469-3.

## Background

Infraocclusion of deciduous teeth is a frequently occurring dental anomaly caused by partial or complete dissolution of Sharpey’s fiber apparatus. Due to fusion of the alveolar bone with the root cementum, affected deciduous teeth do not participate in vertical growth and thus appear increasingly infraoccluded. The cause of ankylosis is still not well understood. Degenerative changes in the periodontium in combination with a genetic background are suspected [1]. The prevalence of infraoccluded deciduous teeth varies significantly from 0.07 to 24.8% in the literature [2–5]. The highest prevalence is described at the ages of 8 and 9 years [1]. While a decrease in the mild form is described with increasing age, there is an increase in the moderate form in 8- to 10-year-olds and the severe form in 11- to 13-year-olds [5].

Infraoccluded deciduous teeth can lead to negative consequences in the long term. Affected teeth sometimes exfoliate late or even require surgical removal [6]. There is a local growth disturbance and an increasing progression of infraocclusion, especially during the puberal growth spurt [7]. The affected teeth may influence the occlusion: there is a risk of overeruption of the antagonist, a shift in the dental midline to the affected side and distal position of the first permanent molar [8, 9]. With pronounced infraocclusion, Becker and Karnei-R’em [10] demonstrated increased tilting and eccentric root movement of neighboring teeth. In addition, neighboring teeth are often undererupted [10]. The extremely early onset of infraocclusion can result in developmental disorders of the successor in the form of malformations, delayed tooth maturation and displacement [11].

A well-founded diagnosis is essential for assessing the development and therapeutic consequences of infraoccluded deciduous teeth. To date, the literature does not present any standardized diagnostic procedures for the quantitative and qualitative assessment of infraoccluded deciduous teeth. The extent of infraposition has been measured very frequently, but reference levels differ [8, 12–14]. Hvaring and Birkeland [15] introduced relative measurements to account for different magnification factors of panoramic radiographs. In contrast, other authors used the semiquantitative classification of infraoccluded deciduous teeth [6, 16, 17]. In addition to dental observations, analyses of the contour of the alveolar ridge [18, 19], the alveolar bone height [10, 11, 20] and the skeletal growth pattern [21, 22] were carried out. Studies on the etiology of infraocclusion of deciduous teeth often use histologic examinations [1, 17, 23].

In addition, numerous secondary parameters for the study of infraoccluded deciduous teeth have been described in the literature. The extent of root resorption [12, 24, 25] and the influence of ankylosis on the tilting of neighboring teeth [12, 26–28] have frequently been analyzed. Other studies investigated the influence of the length of the dental arch [8, 9]. Numerous studies also focused on the formation, position, development and eruption of the successor [6, 11, 20, 25] and its association with other dental anomalies, such as aplasia, supernumerary teeth, microdontia, displaced canines, enamel hypoplasia, taurodontism and odontomas [14, 22, 29, 30].

The aim of this systematic review is to detail the current state of the literature on the qualification and quantification of infraoccluded deciduous teeth. This work focused on assessing the extent of infraocclusion at the occlusal, alveolar and skeletal levels. The establishment of a standardized classification is intended to simplify treatment decisions regarding the extraction or observation of affected deciduous teeth and to improve the evaluation of long-term prognosis.

## Methods

This systematic review was based on the current Preferred Reporting Items for Reporting Systematic Reviews and Meta-Analyses (PRISMA) guidelines [31, 32]. The review has been registered in the international prospective register of systematic reviews (PROSPERO) under the registration number: CRD42024555842. The protocol has not been published. Studies that investigated at least one infraoccluded deciduous tooth per participant quantitatively or qualitatively were included. Histologic investigations and secondary parameters discussing the consequences of infraoccluded deciduous teeth on adjacent teeth, dental arches or successors were not included in the study. Controlled and uncontrolled clinical studies, retrospective studies, observational studies and cross-sectional studies were included. Case reports, case series, comments, expert opinions, letters to the editor, literature reviews and studies enrolling less than 10 subjects or 10 infraoccluded teeth in total were excluded. All articles were limited to English and German languages. An electronic search was conducted on the following databases:

- PubMed (MEDLINE including MEDLINE In-Process) (January 1, 1948 to September 23, 2024);
- Cochrane Central Register of Cochrane Reviews and Controlled Trials (CENTRAL) (January 1, 1992 to September 23, 2024);
- Web of Science Core Collection.
- Science Citation Index Expanded (January 1, 1900 to September 23, 2024);
  - Social Sciences Citation Index (January 1, 1956 to September 23, 2024);
  - Arts & Humanities Citation Index (January 1, 1975 to September 23, 2024);
  - Emerging Sources Citation Index (January 1, 2019 to September 23, 2024);
- Embase.com (Elsevier) (January 1, 1974 to September 23, 2024).

Study registries (ClinicalTrials.gov) were searched (to September 23, 2024), and corresponding authors were contacted if information was missing. The search strategy was developed and monitored by an expert consultant (SW). A detailed list of the search strategy is provided (see Additional file 1). The identified publications were imported into Endnote (version X7.8, Clarivate, Philadelphia, PA), and duplicates were eliminated using the deduplication tool. The remaining duplicates were checked and removed manually. Based on the title and abstract, one investigator (TT) preselected *n* = 156 potentially relevant publications. The findings were independently verified by a second investigator (HKS). Disagreements on *n* = 5 publications were discussed, and a consensus was reached. In the end, *n* = 151 reports were selected for retrieval. A standardized protocol was developed for the analysis of the full texts (see additional file 2). Full-text documents of all relevant papers were analyzed using this protocol by one investigator (TT) and verified by a second investigator (HKS). Disagreements on *n* = 3 publications were discussed, and a joint consensus was reached on the final included publications (*n* = 42). A standardized questionnaire was developed to analyze the study characteristics. Initially, all data from the individual studies were extracted by one investigator (TT) based on the questionnaire (see additional file 3). These were discussed in detail between both investigators (TT, HKS) and a final decision was made based on the inclusion criteria. The following parameters regarding infraoccluded deciduous teeth were extracted in the end (see Table 1):

**Table 1:** Definitions of the individual parameters surveyed. Each column is to be read separately from top to bottom

Study information	Localization	Type of examination	Measurement	Measurement reference	Classification	Method
Author	Affected arch (upper and/or lower arch)	Occlusal offset: Extent of infraposition	Absolute measurement in millimeters or relative measurement	Distance between the occlusal plane (definition according to the author) / adjacent teeth / mesial ridge of the first permanent molar and the infraoccluded deciduous tooth	Infraposition of deciduous teeth classified as mild, moderate and severe according to the author	Clinical examination: Assessment of infraposition by direct inspection
Year		Alveolar ridge: Contour of the alveolar crest				Cast analysis: Measurement infraposition on the plaster model
		Alveolar process: Mandibular height				Radiographic examination: Assessment of infraocclusion by analyzing panoramic radiographs, intraoral radiographs, bitewing views and/or lateral cephalograms
		Skeletal growth pattern: Horizontal, neutral or vertical mandibular growth (according to cephalometric analysis)				

## Results

**Fig. 1:**
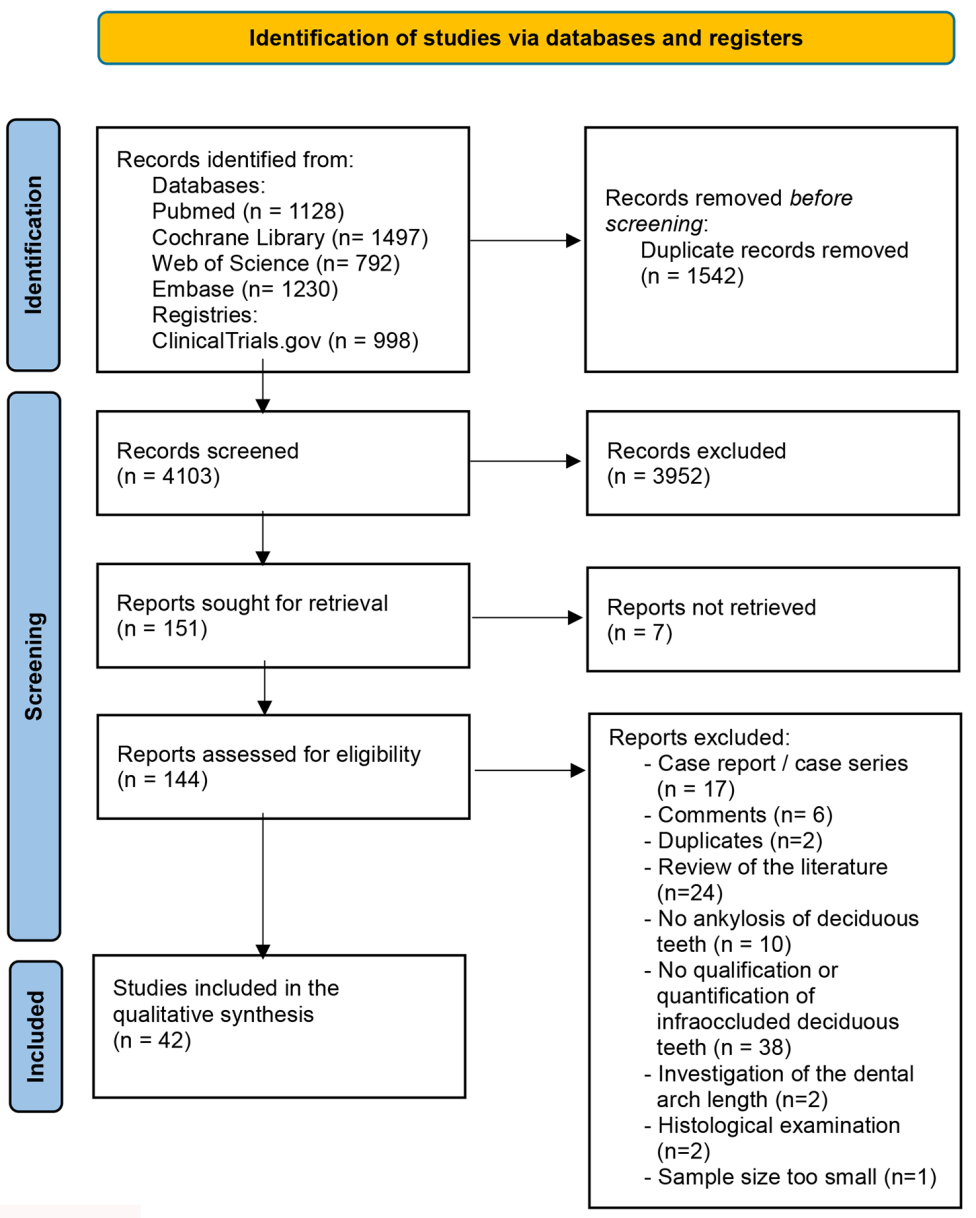
Flow diagram presenting the study selection process according to PRISMA guidelines

Initially, 5645 records were found via an electronic literature search. After duplicate removal, 4103 records were screened, of which 144 full-text documents were reviewed, and 42 papers were ultimately included (see Fig. 1). Table 2 shows the characteristics of the individual studies. Additional file 4 presents a detailed list of the 102 excluded papers.

**Table 2:** Summarized data of the included studies in the review

Author	Year	Localisation	Examination	Measurement	Reference for measurement	Classification of infraocclusion	Method
Rune [23]	1971	upper and lower jaw	occlusal offset	none	distance from the occlusal surface of the infraoccluded deciduous tooth to the occlusal plane (connecting line of the incisal edge of the deciduous canine, the incisors and the cusps of all teeth in occlusion)	none	clinical examination, dental casts
		upper and lower jaw	alveolar ridge	none	none		radiographs (intraoral radiographs)
Brearley, McKibben [16]	1973	upper and lower jaw	occlusal offset			slight: occlusal surface is located approximately 1 mm below the occlusal plane, moderate: occlusal surface is at the height of the contact point of one or both adjacent teeth, severe: occlusal surface is located at the level of or below the gingiva	clinical examination, radiographs (intraoral radiographs)
Darling, Levers [17]	1973	upper and lower jaw	occlusal offset	absolute in millimeters	distance from the occlusal surface of the infraoccluded deciduous tooth to the occlusal plane (connecting line of the cusps or midpoint between lingual and buccal cusps of the adjacent teeth)	(1) slight, (2) marked (3) occlusal surface of tooth only just visible, (4) very deep, (5) very deep and “floating” free in an abscess cavity	dental casts, radiographs (intraoral radiographs)
Sullivan [49]	1976	upper and lower jaw	occlusal offset			according to Darling and Levers, 1973	not specified
Messer, Cline [6]	1980	upper and lower jaw	occlusal offset			slight: occlusal surface of the infraoccluded deciduous tooth is approximately 1 mm below the occlusal plane (closest to non-affected neighboring teeth), moderate: occlusal surface is at the height of the contact point of one or both adjacent teeth, severe: occlusal surface is located at the level of or below the papilla	dental casts
		upper and lower jaw	alveolar ridge	not specified	attachment loss in relation to the height of the alveolar ridge		radiographs (intraoral radiographs)
Brown [46]	1981	upper and lower jaw	skeletal growth pattern		anterior/posterior facial height (perpendicular distance nasion - maxillary plane - menton/gonion), anterior/posterior maxillary height (maxillary plane - menton/gonion)		radiographs (lateral cephalograms), extraoral photographs
Koyoumdjisky-Kaye, Steigman [50]	1982	upper and lower jaw	occlusal offset			according to Brearley and McKibben, 1973	dental casts
Koyoumdjisky-Kaye, Steigman [5]	1982	upper and lower jaw	occlusal offset			according to Brearley and McKibben, 1973	dental casts
Kula et al. [47]	1984	upper and lower jaw	skeletal growth pattern		sella-nasion - sella gnathion angle, sella-nasion - mandibular plane angle		radiographs (lateral cephalograms)
Kurol [1]	1984	upper and lower jaw	occlusal offset	absolute in millimeters	not specified		not specified
Kurol, Thilander [8]	1984	upper and lower jaw	occlusal offset	absolute in millimeters	distance from the occlusal surface of the infraoccluded deciduous tooth to the occlusal plane (connecting line of the mesiobuccal cusp of the first permanent molar and the incisal edge of the central incisors)		dental casts
		lower jaw	alveolar process	absolute in millimeters	distance from the most coronal point of the furcation of the first permanent molar to the inferior border of the mandible		radiographs (panoramic radiographs)
Kurol, Thilander [20]	1984	upper and lower jaw	occlusal offset	absolute in millimeters	distance from the occlusal surface of the infraoccluded deciduous tooth to the occlusal plane (connecting line of the mesiobuccal cusp of the first permanent molar and the incisal edge of the central incisors)		dental casts
		lower jaw	alveolar process	absolute in millimeters	distance from the most coronal point of the furcation of the first permanent molar to the inferior border of the mandible		radiographs (panoramic radiographs)
Kurol, Koch [42]	1985	lower jaw	occlusal offset	absolute in millimeters	distance from the occlusal surface of the infraoccluded deciduous tooth to the occlusal plane (connecting line of the mesiobuccal cusp of the first permanent molar and the incisal edge of the central incisors)		dental casts
		lower jaw	alveolar ridge	not specified	not specified		radiographs (intraoral radiographs)
Kurol, Olson [45]	1991	upper and lower jaw	occlusal offset	not specified	not specified		not specified
		upper and lower jaw	alveolar ridge	absolute in millimeters	distance from the cementoenemal junction mesial to the first permanent molar to the alveolar ridge		radiographs (bitewing radiographs)
Raghoebar et al. [28]	1991	upper and lower jaw	occlusal offset	absolute in millimeters	distance form the occlusal surface of the infraoccluded tooth and the occlusal plane of the affected jaw		clinical examination
		upper and lower jaw	alveolar ridge	not specified	not specified		radiographs (intraoral radiographss, panoramic radiographs)
Becker, Karnei-R’em [26]	1992	lower jaw	occlusal offset	absolute in millimeters	distance from the mindpoint of the occlusal table of the infraoccluded deciduous tooth to the connecting line of the midpoints of the occlusal level of the adjacent teeth		radiographs (panoramic radiographs)
Becker, Karnei-R’em [10]	1992	lower jaw	alveolar process	absolute in millimeters	distance from the center of the occlusal table of the adjacent teeth to the inferior border of the mandible running parallel to the skeletal midline		radiographs (panoramic radiographs)
Bjerklin, Bennett [12]	2000	lower jaw	occlusal offset	absolute in millimeters	distance from the occlusal surface of the infraoccluded deciduous tooth to the connecting line of the cusps of the neighboring teeth		radiographs (intraoral radiographs)
Antoniades et al. [51]	2002	upper and lower jaw	occlusal offset			partial: infraposition of the ankylosed deciduous tooth ≤ 3 mm, severe: infraposition ≥ 3 mm, occlusal surface visible, total: occlusal surface no longer visible, presence of the tooth only detectable on radiograph	radiographs
Quintero et al. [52]	2003	upper and lower jaw	occlusal offset			grade (I) crown of the infraoccluded deciduous tooth is located at the level of the alveolar ridge, grade (II) crown is located below the alveolar ridge	radiographs
Mass et al. [18]	2004	lower jaw	alveolar ridge	in millimeters by pixel analysis	distance from the cementoenemal junction of the infraoccluded deciduous tooth/neighboring teeth/antagonist to the alveolar ridge		radiographs (bitewing radiographs)
Leonardi et al. [22]	2005	lower jaw	skeletal growth pattern		condyle-gnathion angle, condyle-gonion angle, stable basicranial line angle, ramus angle (posterior border of the ramus - condyle), mandibular plane angle (inferior border of the ramus - gnathion)		radiographs (lateral cephalograms)
Bjerklin et al. [53]	2008	lower jaw	occlusal offset	absolute in millimeters	according to Bjerklin and Bennett, 2000		radiographs (dental film, bitewing radiographs)
Kjaer et al. [11]	2008	lower jaw	occlusal offset			mild: occlusal surface of the infraoccluded deciduous tooth is equal to or less than half crown height below the occlusal plane, moderate: occlusal surface is half to full crown height below the occlusal plane, severe: occlusal surface is equal to or more than full crown height below the occlusal plane, extreme: deciduous tooth is deeply subgingival located, occlusal surface is equal to or more than one and a half crown height below the occlusal plane	radiographs (panoramic radiographs, intraoral radiographs)
		lower jaw	alveolar process	absolute in millimeters	shortes distance from the alveolar ridge to the inferior border of the mandible through the midpoint of the mesio-distal collum width of the infraoccluded deciduous tooth		radiographs (panoramic radiographs, intraoral radiographs)
Shalish et al. [14]	2010	upper and lower jaw	occlusal offset	absolute in millimeters	distance from the distal marginal ridge of the infraoccluded deciduous tooth to the mesial marginal ridge of the first permanent molar	mild: 1–2 mm, moderate: >2 mm < 5 mm, severe: >5 mm	dental casts
Silvestrini Biavati et al. [54]	2011	upper and lower jaw	occlusal offset	absolute in millimeters	according to Darling and Levers, 1973	(1) 1–2 mm, (2) 2.5–4 mm, (3) 4.5–9 mm	dental casts
Dias et al. [44]	2012	lower jaw	occlusal offset	absolute in millimeters	distance from the occlusal surface of the infraoccluded deciduous tooth to the occlusal plane (connecting line of the first permanent molar and canine)	mild: occlusal surface of the infraoccluded deciduous tooth is approximately 1 mm below the occlusal plane, moderate: occlusal surface is at the height of or just below the contact point of the adjacent teeth	dental casts, radiographs (panoramic radiographs)
		lower jaw	alveolar process	absolute in millimeters	vertical change of the contour of the alveolar ridge (first permanent molar to canine) within two panoramic radiographs at least 12 months apart		radiographs (panoramic radiographs)
Peretz et al. [19]	2013	lower jaw	occlusal offset	absolute in millimeters	distance from the occlusal level of the infraoccluded deciduous tooth to the occlusal plane (connecting line of the cusps of the neighboring teeth)		radiographs (dental film)
			alveolar ridge	absolute in millimeters	distance from the cementoenemal junction mesial and distal of the infraoccluded deciduous tooth to the alveolar ridge		radiographs (dental film)
Cardoso Silva et al. [24]	2014	lower jaw	occlusal offset	absolute in millimeters	according to Bjerklin and Bennett, 2000 modification: distance from the occlusal surface of the infraoccluded deciduous tooth to the connecting line of the cusps of the neighboring mesial tooth and the most distal and totally erupted permanent first molar or primary second molar	mild: 1.0–1.9 mm, moderate: 2.0–2.9 mm, severe: ≥3 mm	radiographs (panoramic radiographs)
Hvaring et al. [15]	2014	lower jaw	occlusal offset	relative in millimeters	ratio of the crown height of the first permanent molar (y-axis) and the distance from the most prominent distal cusp of the infraoccluded deciduous tooth to the occlusal plane (perpendicular to the y-axis)		radiographs (panoramic radiographs)
Shalish et al. [55]	2014	upper and lower jaw	occlusal offset	absolute in millimeters	vertical distance from the marginal ridge of the infraoccluded deciduous tooth and the marginal ridge of the neighboring first permanent molar		dental casts
Odeh et al. [13]	2015	upper and lower jaw	occlusal offset	absolute in millimeters	distance from the occlusal surface of the infraoccluded deciduous tooth to the occlusal plane (marginal ridge of the first permanent molar to the incisal edge of the deciduous canine)	mild: 1 mm to < 2 mm, moderate: 2 mm to < 3 mm, severe: ≥3 mm	dental casts, radiographs (panoramic radiographs)
Odeh et al. [43]	2016	upper and lower jaw	occlusal offset	absolute in millimeters	according to Odeh et al., 2015	according to Odeh et al., 2015	dental casts, radiographs (panoramic radiographs)
Lanteri et al. [21]	2020	lower jaw	occlusal offset	not specified	distance from the occlusal surface of the infraoccluded deciduous tooth to the occlusal plane (connecting line from the most mesial point of the first permanent molar to the canine)		radiographs (panoramic radiographs)
		lower jaw	skeletal growth pattern		distance supraorbital point - spina nasalis anterior, distance spina nasalis anterior - menton, gonional angle (upper/lower/total), condyle position, mandibular shape		radiographs (lateral cephalograms)
Ciftic et al. [29]	2021	upper and lower jaw	occlusal offset			according to Brearley and McKibben, 1973	radiographs (panoramic radiographs)
Alshaya et al. [56]	2022	upper and lower jaw	occlusal offset			according to Brearley and McKibben, 1973	radiographs (panoramic radiographs)
Calheiros-Lobo et al. [41]	2022	lower jaw	occlusal offset	absolute in millimeters	distance from the occlusal surface of the infraoccluded deciduous tooth to the occlusal plane (connecting line of the cusps of the neighboring teeth)		radiographs (panoramic radiographs)
Eşian et al. [57]	2022	upper and lower jaw	occlusal offset			mild: infraposition of the infraoccluded deciduous tooth < 1 mm, moderate: infraposition > 1 mm, severe: occlusal surface below the contact point of adjacent teeth	radiographs (panoramic radiographs)
Renugalakshmi et al. [30]	2023	upper and lower jaw	occlusal offset	absolute in millimeters	according to Odeh et al., 2015		radiographs (panoramic radiographs)
Akgöl et al. [38]	2024	upper and lower jaw	occlusal offset	absolute in millimeters	distance from the occlusal surface of the infraoccluded deciduous tooth to the occlusal plane (connecting line of the cusps of the neighboring teeth)	according to Kjaer et al. 2008	radiographs (panoramic radiographs)
Ishizuka et al. [58]	2024	upper and lower jaw	occlusal offset	absolute in millimeters	distance from the occlusal surface of the infraoccluded deciduous tooth to the occlusal plane (connecting line of the cusps of the neighboring teeth); in the case of an angulation of the adjacent teeth a line was drawn between the tips of the highest cusps		radiographs (panoramic radiographs)
Marcianes et al. [59]	2024	lower jaw	occlusal offset	absolute in millimeters	according to Odeh et al., 2015, in the case of lacking the ipsilateral deciduous canine according to Cardoso et al., 2014		radiographs (panoramic radiographs)

## Discussion

Various treatment decision models have been introduced in the literature, which are primarily based on the degree of infraocclusion (mild, moderate, severe), root resorption level, tilting of the adjacent teeth and agenesis or no agenesis of the successor [33–35]. However, the degree of infraocclusion alone is not always associated with the severity of ankylosis or its effect on the alveolar process [11]. Furthermore, studies have shown a lack of correlation between root resorption and the long-term prognosis of deciduous teeth [36, 37]. Whereas tilting of the adjacent teeth is a secondary dental effect [26]. The development of the successor, however, is decisive in the evaluation of infraoccluded deciduous teeth. Normal exfoliation of affected teeth with a successor is likely without the need for treatment intervention [8]. Exfoliation of affected teeth without a successor is unlikely and a more pronounced infraposition is expected instead [38]. Infraoccluded teeth without successor are therefore critical to assess.

**Fig. 2:**
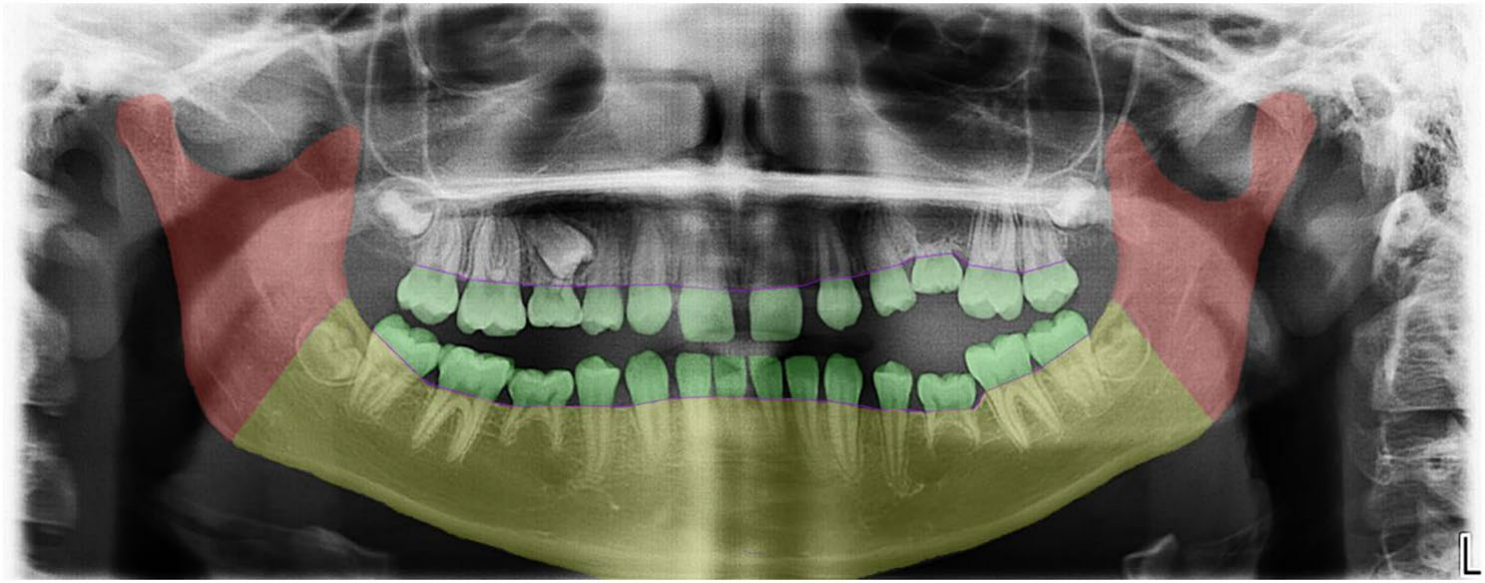
Visualization of the assessment of infraoccluded deciduous teeth using a stage model: While the color green indicates the assessment of infraoccluded deciduous teeth at the occlusal level (= occlusal offset), the color purple reflects the assessment of the contour of the alveolar crest (= alveolar ridge), the color yellow the height of the mandible (= alveolar process), and red the vertical mandibular growth pattern initiated by condylar growth (= skeletal growth pattern)

In all treatment decision making models growth is not taken into account. The increase of the infraposition of infraoccluded deciduous teeth correlates with the rate of growth [17]. The evaluation of the extent of growth inhibition is therefore important for an appropriate therapeutic approach, in addition to the consideration of the development of the successor. In the present study, the focus therefore was placed on the various assessment levels of infraoccluded deciduous teeth, with particular attention to the influence on alveolar growth (see Fig. 2). In the vast majority of studies (*n* = 37), affected teeth were assessed at the occlusal level (see Table 2). Significantly fewer studies assessed infraoccluded deciduous teeth at the level of the alveolar ridge (*n* = 7) or the alveolar process (*n* = 5). Qualitative examination of the vertical skeletal growth pattern was performed even less frequently (*n* = 4).

**Fig. 3:**
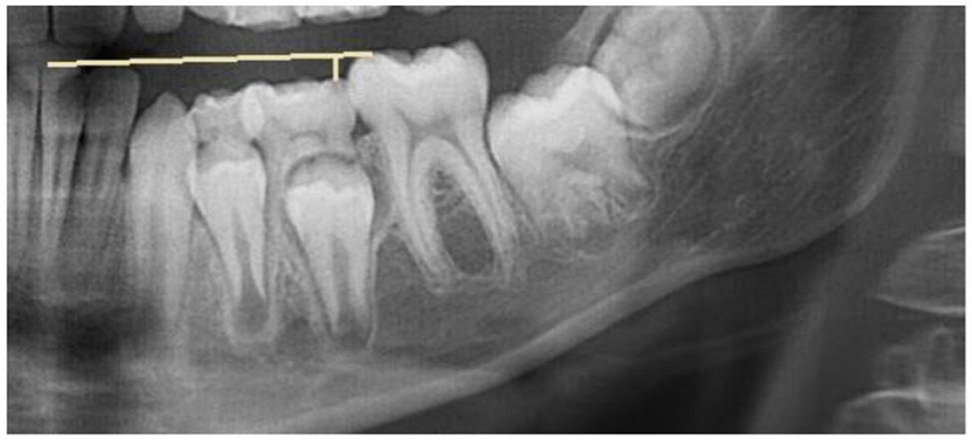
Determination of the extent of infraocclusion according to Kurol and Thilander [8] by measuring the shortest distance from the ankylosed deciduous tooth to the occlusal plane (the mesiobuccal cusp of the first permanent molar to the incisal edge of the incisors)

**Fig. 4:**
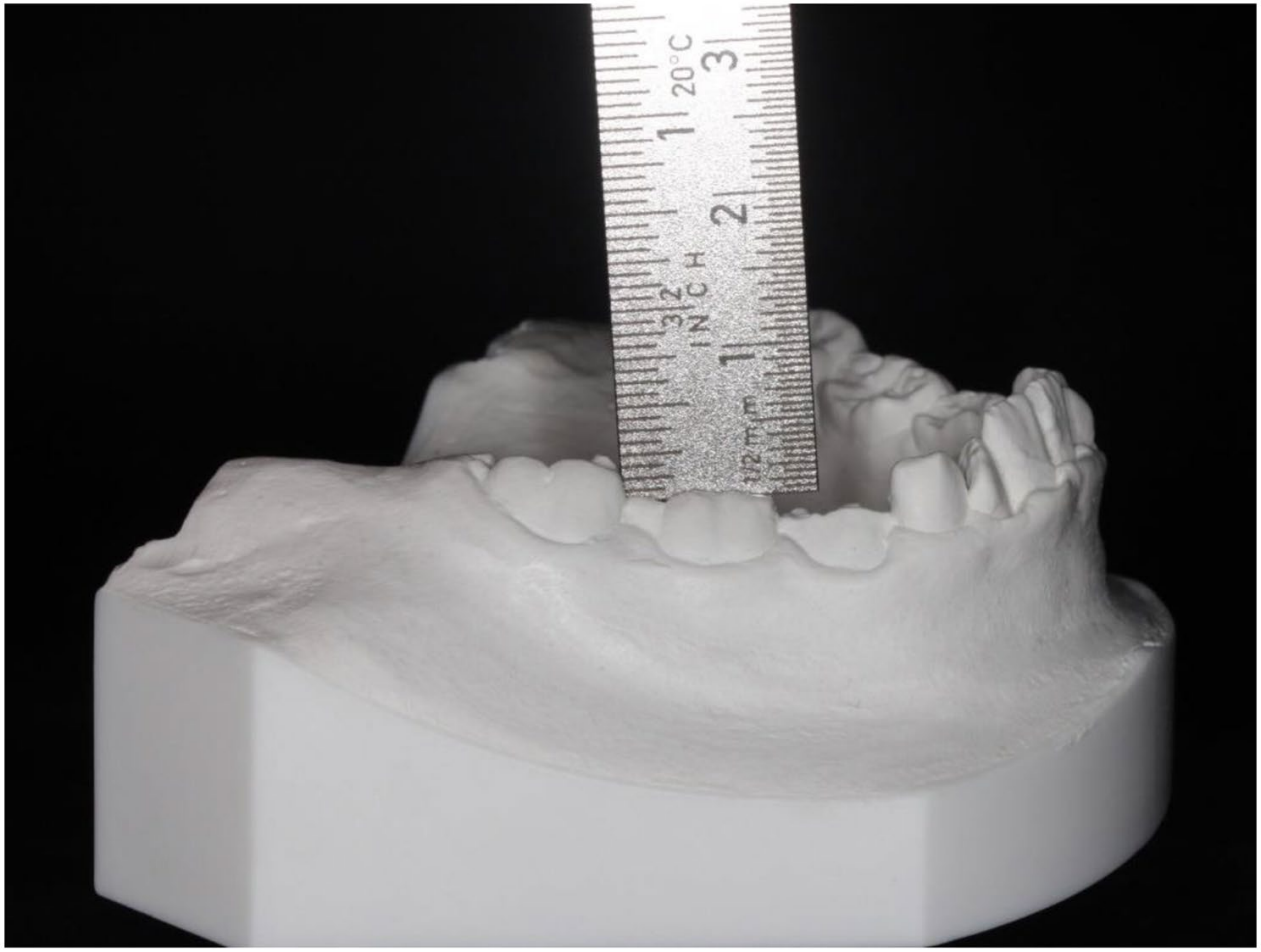
Determination of the extent of infraocclusion according to Shalish et al. [14] by measuring the shortest vertical distance from the distal marginal ridge of the ankylosed deciduous tooth to the mesial marginal ridge of the first permanent molar. The second deciduous molar is 2,5 mm infraoccluded

**Fig. 5:**
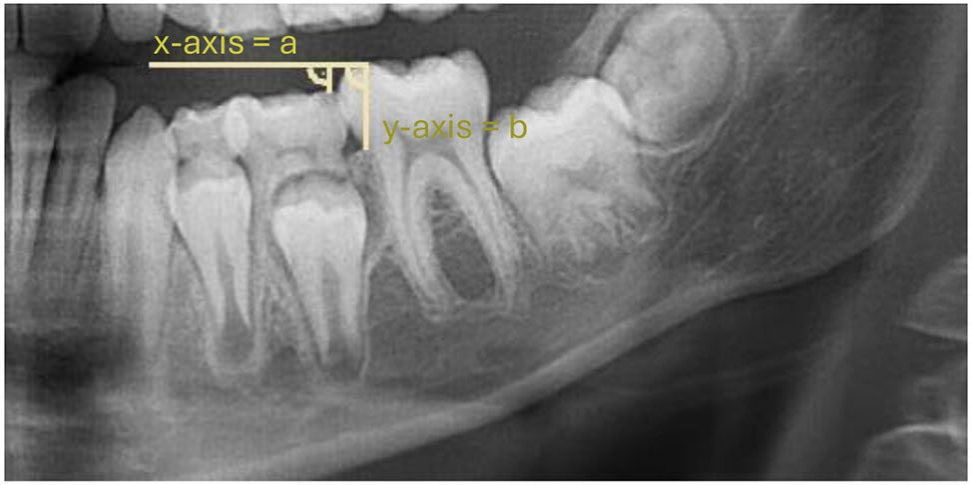
Relative measurement of the infraocclusion of an ankylosed second deciduous molar according to Hvaring and Birkeland [36]. The y-axis is defined as the distance b: distance from the mesial cementoenamel junction to the mesial cusp tip of the first permanent molar. The x-axis runs perpendicular to the y-axis. Infraocclusion is presented as the ratio (R = a/b), which describes the ratio of the shortest distance from the most prominent distal cusp of the ankylosed deciduous tooth to the x-axis (distance a) to the crown height of the first permanent molar (distance b)

The occlusal offset was mainly evaluated by metric measurements (*n* = 23). Fewer studies assessed the extent of infraocclusion using semiquantitative categories (*n* = 13). *N* = 1 study investigated the extent of the infraposition of deciduous teeth exclusively by clinical examination [23]. The reason given by the author was the variability of the occlusal plane as a reference plane, which depends on the inclination of individual teeth, the extent of attrition, the curve of spee, the eruption stage and the number of teeth in the quadrant. The advantage of categorization lies mainly in the ease of use and the subordinate role of standardization of radiographs. However, the nature of the ordinal scale must be taken into account in the statistical analysis [39]. In addition, the categorization is based on subjective observations, therefore a number of studies used categories based on previously performed metric measurements (*n* = 5). With the exception of one study by Hvaring and Birkeland [15], the metric measurements were always absolute measurements (see Figs. 3 and 4). The advantage can be attributed to its simple application and statistical evaluation. However, the reference levels differed between the studies (see Table 3). This makes it much more difficult to compare the measured values from different studies. In addition to the limitations imposed by the variability of the occlusal plane as a reference plane, the use of panoramic radiographs is compromised by the effects of magnification, distortion, and motion artifacts. Consequently, Hvaring and Birkeland [15] introduced the relative measurement of infraocclusion (see Fig. 5). However, the dependence on the crown length of the first permanent molar and the difficulty in marking the cementoenamel junction should be noted [40]. Becker and Karnei-R’em [10] found the first permanent molar adjacent to an ankylosed deciduous tooth to be an unsuitable reference due to evidence of undereruption. While older studies prior to the year 2000 primarily used non-radiographic methods such as clinical examination or cast analysis to determine the extent of infraocclusion of deciduous teeth at the occlusal level, the use of radiographic methods increased significantly in studies after the year 2000.

**Table 3:** Overview of several definitions of the occlusal plane as a reference plane for evaluating the extent of infraoccluded deciduous teeth used in scientific studies

- Connecting line of the incisal edge of the deciduous canine, the incisors and the cusps of all teeth in occlusion [23]
- Connecting line of the cusps of the neighboring teeth [12, 17, 19, 24, 41]
- Connecting line of the occlusal surface of the neighboring teeth [17, 26]
- Connecting line of the mesiobuccal cusp of the first permanent molar to the incisal edge of the central incisors [8, 20, 42]
- Connecting line of the marginal ridge of the first permanent molar to the incisal edge of the canine [13, 21, 43, 44]

**Fig. 6:**
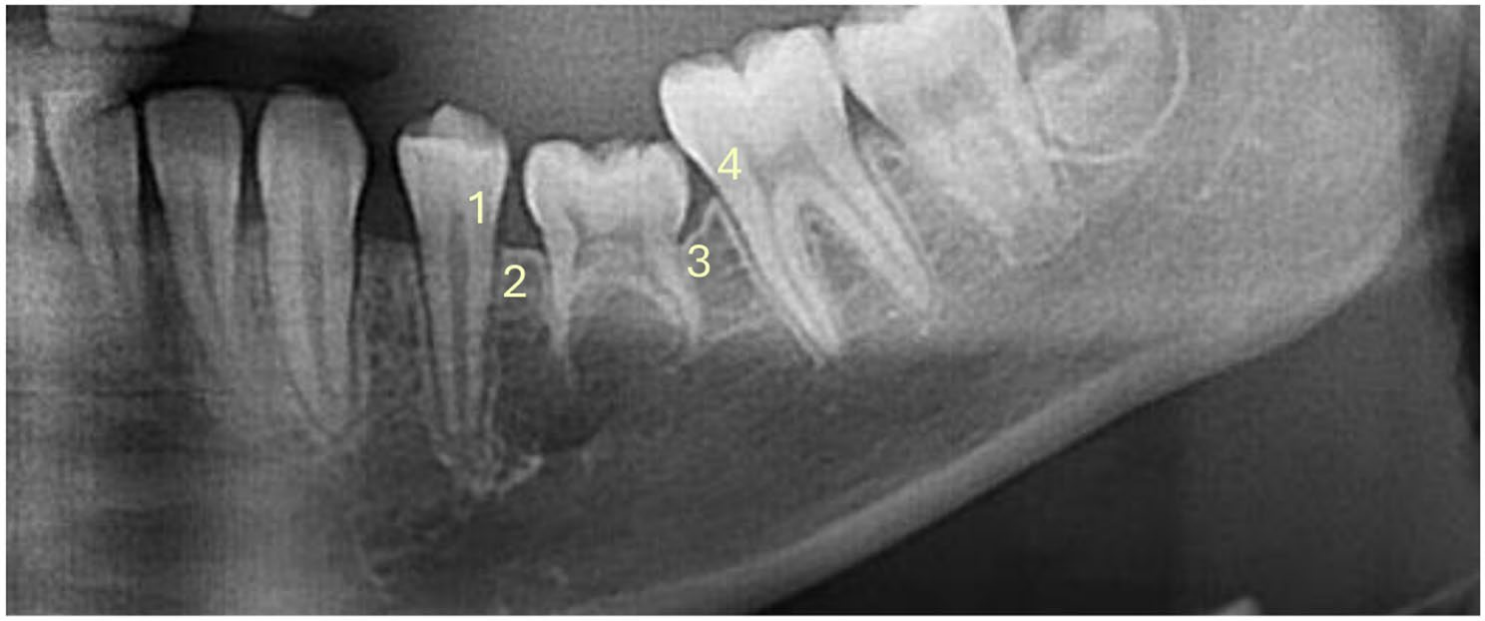
Assessment of the distance of the cementoenemal junction to the alveolar ridge according to Mass et al. [18]: 1 = distal surface of the adjacent mesial tooth, 2 = mesial surface of the infraoccluded tooth, 3 = distal surface of the infraoccluded tooth, 4 = mesial surface of the adjacent distal tooth

The analysis of the alveolar ridge is a radiologic method for the evaluation of infraoccluded deciduous teeth, in which intraoral images (intraoral films, bitewing images) and panoramic radiographs are used. When examining the contour of the alveolar ridge, Kurol and Koch [42] found that infraoccluded deciduous teeth in situ showed a concave curve that normalized after exfoliation. However, other authors have focused on the distance from the cementoenemal junction to the alveolar bone ridge. Mass et al. [18] detected a reduced distance in affected deciduous teeth (see Fig. 6). Kurol and Olson [45] evaluated the attachment loss of the first permanent molar five years after exfoliation or extraction of infraoccluded second deciduous molars. In accordance with Peretz et al. [19], the authors found no association between the extent of infraocclusion and alveolar bone loss. In contrast, Messer and Cline [6] detected a risk for successors in terms of underdevelopment of the alveolar bone and periodontal pocket formation. Particularly premolars associated with retained infraoccluded deciduous teeth and infraoccluded deciduous teeth requiring extraction were affected.

**Fig. 7:**
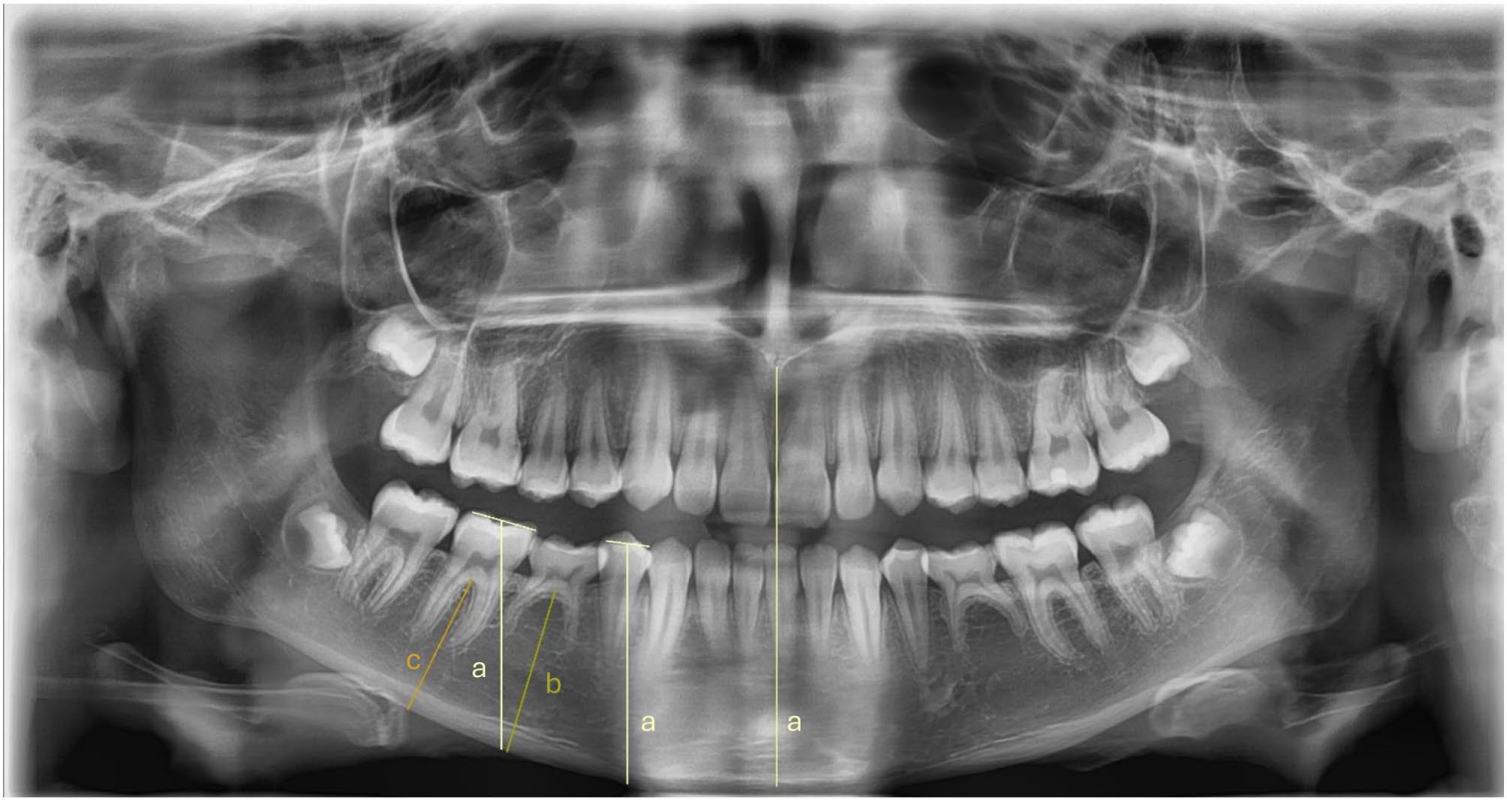
Determination of the alveolar bone height by **a**) measuring the distance from the center of the occlusal surface of the adjacent teeth to the inferior border of the mandible, parallel to the skeletal midline according to Becker and Karnei-R’em [10], **b**) measuring the shortest distance from the alveolar ridge to the inferior border of the mandible through the midpoint of the cervical mesio-distal diameter of the ankylosed deciduous tooth according to Kjaer et al. [11], and **c**) measuring the shortest distance from the most coronal point of the furcation of the first permanent molar to the inferior border of the mandible according to Kurol and Thilander [20]

Studies that analyzed the alveolar bone height using panoramic radiographs showed an inhibition of vertical growth in the region of infraoccluded deciduous teeth and adjacent teeth (see Fig. 7). Kjaer et al. [11] observed a reduction in alveolar bone height in the region of affected deciduous teeth with increasing infraocclusion, except for patients with extremely early onset and therefore severely affected teeth. Dias et al. [44] also confirmed a reduction in vertical alveolar bone growth in the first permanent molars and second premolar region over time using a subtraction method. Deviations were detected by the superimposition of two panoramic radiographs with a minimum time interval of one year using the inferior border of the mandible and the mesiodistal width of the first permanent molar as fixed points. Becker and Karnei-R’em [10] examined in detail the undereruption of neighboring teeth in combination with the change in inclination of the neighboring teeth. Kurol and Thilander [20] compared the alveolar bone height of bilateral infraoccluded deciduous teeth with unilateral aplasia and found no difference in height. All studies analyzing alveolar bone height were limited to the lower arch only. No study assessed the influence of infraoccluded deciduous teeth on vertical growth in the upper arch.

**Fig. 8:**
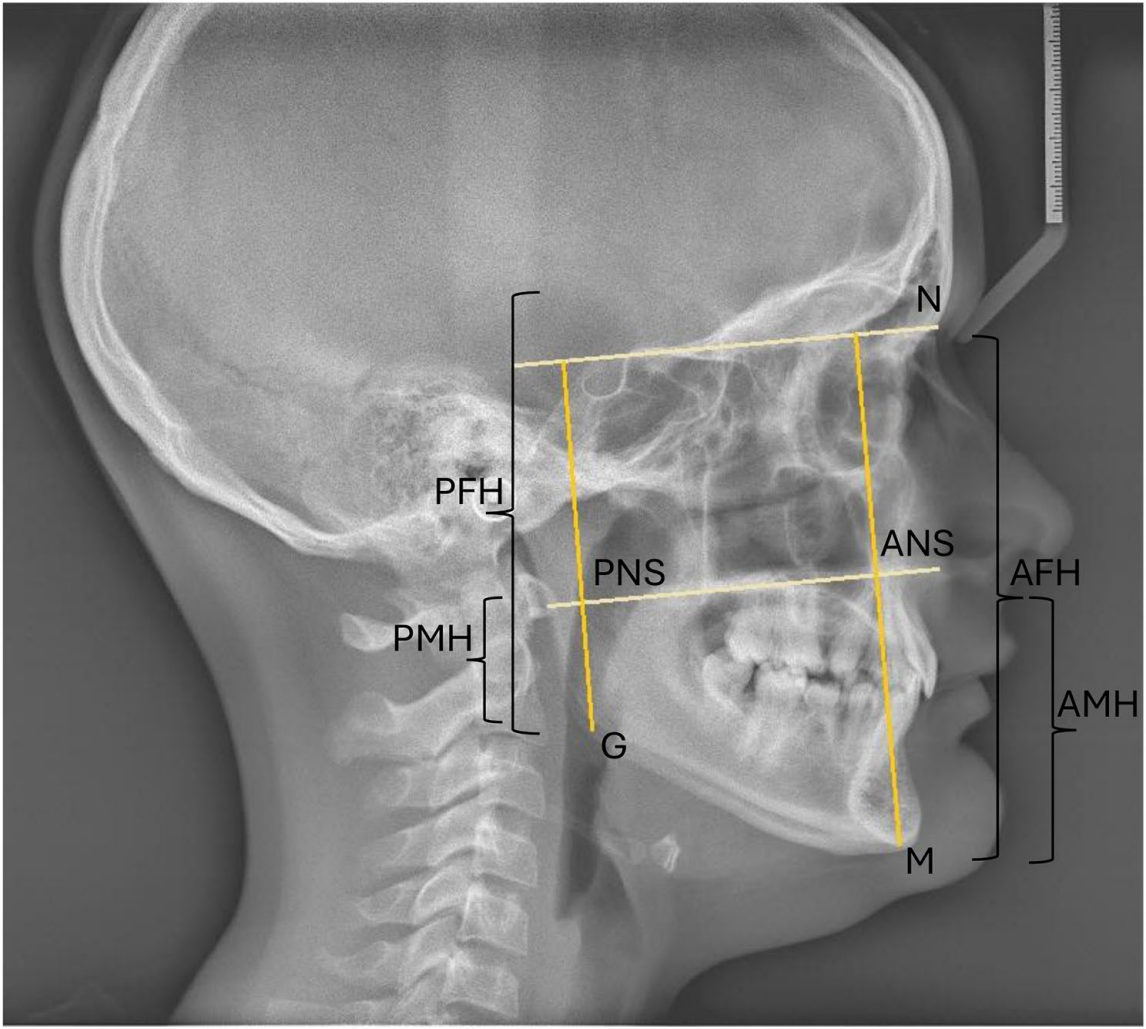
Cephalometric analysis showing the landmarks (N = nasion, G = gonion, M = menton, ANS = anterior nasal spine, PNS = posterior nasal spine) and construction lines (AFH = anterior facial height, PFH = posterior facial height, AMH = anterior maxillary height, PMH = posterior maxillary height) according to Brown [46]

A more far-reaching, qualitative approach to the evaluation of infraoccluded deciduous teeth was chosen by Brown [46], Kula et al. [47], Leonardi et al. [22] and Lanteri et al. [21]. The authors examined vertical skeletal parameters to identify skeletal growth characteristics. The studies reveal contrasting findings. While Leonardi et al. [22] found significant anterior rotation of the mandible in patients with affected deciduous teeth in a longitudinal study, the latter authors indicated a possible posterior rotating mandibular growth tendency. Brown [46], on the other hand, found no significant difference between patients with infraoccluded deciduous teeth and a control group in terms of the ratio of anterior to posterior facial height (see Fig. 8). Kula et al. [47], however, found a tendency towards a larger anterior than posterior facial growth in patients with infraoccluded deciduous teeth. A vertical growth pattern is associated with increased alveolar remodeling [48]. An increase in the infraposition would therefore be expected. The growth pattern may therefore be important in predicting the development of infraocclusion of deciduous teeth. However, the discrepancy in the results does not confirm this. The heterogeneity may be due to the study design and the different use of the cephalometric analysis.

In conclusion, there is a lack of standardization in the analysis of the quantitative and qualitative assessment of infraoccluded deciduous teeth in scientific studies. This is the first scientific paper to assess affected deciduous teeth using a stepwise model at the occlusal, alveolar and skeletal levels. Nevertheless, limitations of the results must be taken into account due to the inclusion of studies with small cohorts. Due to time constraints, single pre-screening was performed for titles and abstracts. In addition, one author performed data extraction. A second reviewer verified the data. Because these steps were not performed by two independent reviewers, some risk of error may be introduced. In addition, the literature search was limited to publications to English and German languages. However, the methodological limitations are not expected to influence the conclusions of this review. The analysis shows that a single assessment of the occlusal level is not sufficient for a comprehensive evaluation of the extent and progression of infraoccluded deciduous teeth. The assessment of the alveolar ridge is essential due to the influence of ankylosis on the transseptal fiber arrangement and the associated changes in the contour of the alveolar ridge and the position of the adjacent teeth [26]. The alveolar process plays a significant role in assessing the influence of local disturbances of the periodontal ligament on growth. Clinically, this means that infraoccluded deciduous teeth with signs of alveolar growth retardation should be viewed critically. Especially in the growth phase, treatment in the form of extraction may be favored, particularly in cases without a successor. The thorough diagnosis of affected deciduous teeth is therefore of particular importance to paediatric dentists and orthodontists. In the future, treatment decision models should be expanded by clinically relevant parameters such as the assessment of alveolar bone growth. The benefit of evaluating the vertical skeletal growth pattern by cephalometric radiographs has not yet been clarified. Future research is needed.

## Conclusions

- Infraoccluded deciduous teeth affect both occlusion and vertical alveolar growth by influencing the surrounding tissues.
- Most studies focus exclusively on the extent of infraposition when investigating infraoccluded deciduous teeth. The influence on alveolar and skeletal vertical growth is given less consideration.
- There are no studies that have investigated the relation between infraoccluded deciduous teeth and alveolar growth in the upper arch.
- The use of a stage model was implemented, which considers (1) occlusal extent of infraposition, (2) contour of the alveolar ridge, (3) height of alveolar process and (4) skeletal vertical growth pattern.
- Clinical examination, cast analysis and panoramic radiographs are essential for a comprehensive diagnosis of infraoccluded deciduous teeth. Intraoral films are inappropriate due to missing representation of the alveolar process.
- The development of standardized, objective parameters is necessary. This primarily concerns methods for evaluating the influence of infraoccluded deciduous teeth on alveolar bone growth in the upper arch.
- When growth inhibition has occurred, extraction of the affected tooth is suggested, particularly during the growth phase and in cases without a successor.

## Electronic supplementary material

Below is the link to the electronic supplementary material.

Supplementary Material 1

Supplementary Material 2

Supplementary Material 3

Supplementary Material 4

## Data Availability

No datasets were generated or analysed during the current study.

## Abbreviations

PRISMA: Preferred Reporting Items on Systematic Reviews and Meta-analysis
PROSPERO: International prospective register of systematic reviews
