# Supplementary material for: Qualitative and quantitative assessment of infraoccluded deciduous teeth: a systematic review
Source: Head Face Med. 2024 Oct 30;20:65. doi: 10.1186/s13005-024-00469-3 (PMC11524026; doi:10.1186/s13005-024-00469-3)
Supplement: Supplementary file 1 — Supplementary Material 1 [file 13005_2024_469_MOESM1_ESM.pdf]

Additional file 1 Results of the initial search. Database search conducted on September 23, 2024.

PubMed (MEDLINE including MEDLINE In-Process)

	String	Results
1	Tooth, Deciduous[Mesh] OR deciduous tooth[tiab] OR deciduous teeth[tiab] OR deciduous dentition*[tiab] OR primary dentition*[tiab] OR milk tooth[tiab] OR primary teeth[tiab] OR primary tooth[tiab] OR deciduous teeth[tiab] OR milk teeth[tiab] OR baby teeth[tiab] OR baby tooth[tiab] OR primary molar*[tiab]	19,701
2	Tooth, Impacted[Mesh] OR impacted tooth[tiab] OR impacted teeth[tiab] OR impaction*[tiab] OR retention[tiab] OR embedded[tiab] OR infra*[tiab] OR submer*[tiab] OR reimpaction*[tiab] OR reinclusion*[tiab]	740,250
3	alveolar bone height*[tiab] OR alveolar bone level*[tiab] OR Alveolar Process[Mesh] OR alveolar process*[tiab] OR alveolar ridge*[tiab] OR occlusal plane*[tiab] OR Vertical Dimension[Mesh] OR vertical dimension*[tiab] OR mandibular rest position*[tiab] OR vertical position*[tiab] OR vertical deficienc*[tiab] OR tooth movement*[tiab] OR jaw growth*[tiab] OR facial growth*[tiab] OR facial development*[tiab]	36,918
4	Tooth Ankylosis[Mesh] OR ankylos*[tiab]	25,343
5	case reports[pt]	2,433,686
6	german[la] OR english[la]	33,817,648
7	1 AND (2 OR 3 OR 4) AND 6 NOT 5	1,128

Cochrane Library

	String	Results
1	[mh "Tooth, Deciduous"] OR<br>("deciduous tooth"):ti,ab,kw OR<br>("deciduous teeth"):ti,ab,kw OR<br>(deciduous dentition*):ti,ab,kw OR<br>(primary dentition*):ti,ab,kw OR<br>("milk tooth"):ti,ab,kw OR<br>("primary teeth"):ti,ab,kw OR<br>("primary tooth"):ti,ab,kw OR<br>("deciduous teeth"):ti,ab,kw OR<br>("milk teeth"):ti,ab,kw OR<br>("baby teeth"):ti,ab,kw OR<br>("baby tooth"):ti,ab,kw OR<br>(primary molar*):ti,ab,kw	6,752
2	[mh "Tooth, Impacted"] OR<br>("impacted tooth"):ti,ab,kw OR<br>("impacted teeth"):ti,ab,kw OR<br>(impaction*):ti,ab,kw OR<br>("retention"):ti,ab,kw OR<br>("embedded"):ti,ab,kw OR<br>(infra*):ti,ab,kw OR<br>(submer*):ti,ab,kw OR<br>(reimpaction*):ti,ab,kw OR<br>(reinclusion*):ti,ab,kw	54,129
3	(alveolar bone height*):ti,ab,kw OR	8,938

	(alveolar bone level*):ti,ab,kw OR<br>[mh "Alveolar Process"] OR<br>(alveolar process*):ti,ab,kw OR<br>(alveolar ridge*):ti,ab,kw OR<br>(occlusal plane*):ti,ab,kw OR<br>[mh "Vertical Dimension "] OR<br>(vertical dimension*):ti,ab,kw OR<br>(mandibular rest position*):ti,ab,kw OR<br>(vertical position*):ti,ab,kw OR<br>(vertical deficienc*):ti,ab,kw OR<br>(tooth movement*):ti,ab,kw OR<br>(jaw growth*):ti,ab,kw OR<br>(facial growth*):ti,ab,kw OR<br>(facial development*):ti,ab,kw	
4	[mh "Tooth Ankylosis"] OR<br>(ankylos*):ti,ab,kw	3,050
5	1 AND (2 OR 3 OR 4)	1,497

Web of Science

	String	Results
1	TS="deciduous tooth" OR<br>TS="deciduous teeth" OR<br>TS="deciduous dentition*" OR<br>TS="primary dentition*" OR<br>TS="milk tooth" OR<br>TS="primary teeth" OR<br>TS="primary tooth" OR<br>TS="deciduous teeth" OR<br>TS="milk teeth" OR<br>TS="baby teeth" OR<br>TS="baby tooth" OR<br>TS="primary molar*"	11,541
2	TS="impacted tooth*" OR<br>TS="impacted teeth*" OR<br>TS="impaction*" OR<br>TS="retention" OR<br>TS="embedded" OR<br>TS="infra*" OR<br>TS="submer*" OR<br>TS="reimpaction*" OR<br>TS="reinclusion*" OR	1,974,659
3	TS="alveolar bone height*" OR<br>TS="alveolar bone level*" OR<br>TS=alveolar Process OR<br>TS="alveolar Process*" OR<br>TS="alveolar Ridge*" OR	35,179

	TS="occlusal plane*" OR<br>TS="vertical Dimension*" OR<br>TS="mandibular rest position*" OR<br>TS="vertical position*" OR<br>TS="vertical deficienc*" OR<br>TS="tooth movement*" OR<br>TS="jaw growth*" OR<br>TS="facial growth*" OR<br>TS="facial development*"	
4	TS="ankylos*"	32,786
5	TI="case report*" OR<br>AB="case report*"	425,662
6	LA=german OR<br>LA=english	76,933,037
7	1 AND (2 OR 3 OR 4) AND 6 NOT 5	792

Embase.com (Elsevier)

	String	Results
1	'deciduous tooth'/exp OR 'deciduous tooth':ab,ti,kw OR 'deciduous dentition*':ab,ti,kw OR 'primary dentition*':ab,ti,kw OR 'milk tooth':ab,ti,kw OR 'primary teeth':ab,ti,kw OR 'primary tooth':ab,ti,kw OR 'deciduous teeth':ab,ti,kw OR 'milk teeth':ab,ti,kw OR 'baby teeth':ab,ti,kw OR 'baby tooth':ab,ti,kw OR 'primary molar*':ab,ti,kw	20,013
2	'tooth impaction'/exp OR 'impacted tooth':ab,ti,kw OR 'impacted teeth':ab,ti,kw OR 'impaction*':ab,ti,kw OR 'retention':ab,ti,kw OR 'embedded':ab,ti,kw OR 'infra*':ab,ti,kw OR 'submer*':ab,ti,kw OR 'reimpaction*':ab,ti,kw OR 'reinclusion*':ab,ti,kw	875,098
3	'alveolar bone height*':ab,ti,kw OR 'alveolar bone level*':ab,ti,kw OR 'alveolar bone'/exp OR 'alveolar process*':ab,ti,kw OR 'alveolar ridge*':ab,ti,kw OR 'occlusal plane*':ab,ti,kw OR 'vertical dimension of occlusion'/exp OR 'vertical dimension*':ab,ti,kw OR 'mandibular rest position*':ab,ti,kw OR 'vertical position*':ab,ti,kw OR 'vertical deficienc*':ab,ti,kw OR 'tooth movement*':ab,ti,kw OR 'jaw growth*':ab,ti,kw OR 'facial growth*':ab,ti,kw OR 'facial development*':ab,ti,kw	29,451
4	'ankylos*':ab,ti,kw	38,315
5	'case report*':ab,ti,kw	719,369
6	([english]/lim OR [german]/lim)	40,453,327
7	1 AND (2 OR 3 OR 4) AND 6 NOT 5	1,230

ClinicalTrials.gov *(https://www.clinicaltrials.gov/ct2/results/refine?show_xprt=Y)*

from database inception until December 14, 2023

	**String**	**Results**
1	(Deciduous Tooth OR Deciduous Dentition OR Primary Dentition OR Milk Tooth OR Primary Teeth OR Primary Tooth OR Deciduous Teeth OR Milk Teeth OR Baby Teeth OR Baby Tooth OR primary molar OR primary molars) AND (Impacted Tooth OR Impacted Teeth OR impaction OR retention OR embedded OR infraocclusion OR infraposition OR infraerupted OR submergence OR submersion OR Reimpaction OR reinclusion)	641
2	(Deciduous Tooth OR Deciduous Dentition OR Primary Dentition OR Milk Tooth OR Primary Teeth OR Primary Tooth OR Deciduous Teeth OR Milk Teeth OR Baby Teeth OR Baby Tooth OR primary molar) AND (alveolar bone height OR alveolar bone level OR Alveolar Process OR Alveolar Process OR Alveolar Ridge OR Occlusal plane OR Occlusal plane OR Vertical Dimension OR Vertical Dimension OR Mandibular Rest Position OR vertical position OR vertical deficiency OR tooth movement OR jaw growth OR Facial growth OR Facial development)	352
3	(Deciduous Tooth OR Deciduous Dentition OR Primary Dentition OR Milk Tooth OR Primary Teeth OR Primary Tooth OR Deciduous Teeth OR Milk Teeth OR Baby Teeth OR Baby Tooth OR primary molar) AND (tooth ankylosis OR dental ankylosis OR ankylosed teeth)	3

ClinicalTrials.gov *(https://www.clinicaltrials.gov/)*

from December 15, 2023 until September 23, 2024

	**String**	**Results**
1	(Deciduous Tooth OR Deciduous Dentition OR Primary Dentition OR Milk Tooth OR Primary Teeth OR Primary Tooth OR Deciduous Teeth OR Milk Teeth OR Baby Teeth OR Baby Tooth OR primary molar OR primary molars) AND (Impacted Tooth OR Impacted Teeth OR impaction OR retention OR embedded OR infraocclusion OR infraposition OR infraerupted OR submergence OR submersion OR Reimpaction OR reinclusion)	2
2	(Deciduous Tooth OR Deciduous Dentition OR Primary Dentition OR Milk Tooth OR Primary Teeth OR Primary Tooth OR Deciduous Teeth OR Milk Teeth OR Baby Teeth OR Baby Tooth OR primary molar) AND (alveolar bone height OR alveolar bone level OR Alveolar Process OR Alveolar Process OR Alveolar Ridge OR Occlusal plane OR Occlusal plane OR Vertical Dimension OR Vertical Dimension OR Mandibular Rest Position OR vertical position OR vertical deficiency OR tooth movement OR jaw growth OR Facial growth OR Facial development)	0
3	(Deciduous Tooth OR Deciduous Dentition OR Primary Dentition OR Milk Tooth OR Primary Teeth OR Primary Tooth OR Deciduous Teeth OR Milk Teeth OR Baby Teeth OR Baby Tooth OR primary molar) AND (tooth ankylosis OR dental ankylosis OR ankylosed teeth)	0
