# Supplementary material for: Qualitative and quantitative assessment of infraoccluded deciduous teeth: a systematic review
Source: Head Face Med. 2024 Oct 30;20:65. doi: 10.1186/s13005-024-00469-3 (PMC11524026; doi:10.1186/s13005-024-00469-3)
Supplement: Supplementary file 2 — Supplementary Material 2 [file 13005_2024_469_MOESM2_ESM.pdf]

Additional file 2 Full text analysis.

Date:

Titel:

Author:

Publication year:

Study design

Prospective study ☐

Retrospective study ☐

Cross-sectional study ☐

Observational study ☐

Clinical study ☐

Histological study ☐

Literature review ☐ n=

Number of patients included: n=

Number of patients affected: n=

Number of teeth affected: n=

One-time finding ☐

Progression ☐

Reference:

Quantification ☐

Qualification ☐

Radiography ☐

Panoramic radiograph ☐

Intraoral radiograph ☐

Bitewing view ☐

Lateral cephalogram ☐

Photographs ☐

Dental cast ☐

Clinical investigation ☐

What type?

Dental anomaly ☐
