# Supplementary material for: Qualitative and quantitative assessment of infraoccluded deciduous teeth: a systematic review
Source: Head Face Med. 2024 Oct 30;20:65. doi: 10.1186/s13005-024-00469-3 (PMC11524026; doi:10.1186/s13005-024-00469-3)
Supplement: Supplementary file 3 — Supplementary Material 3 [file 13005_2024_469_MOESM3_ESM.pdf]

Additional file 3 Questionnaire for data extraction

<table>
<tr><th>Author</th><th>Publication year</th><th>Localisation infraoccluded deciduous teeth</th><th>Type of examination</th><th>Measure ment</th><th>Reference for measurement</th><th>Method</th></tr>
<tr><td rowspan="4"></td><td rowspan="4"></td><td rowspan="4">Upper jaw ☐
Lower jaw ☐</td><td>occlusal offset ☐</td><td rowspan="4">Absolute ☐
Relative ☐</td><td>Occlusal plane ☐
Definition:
____
Adjacent teeth ☐
Mesial ridge first permanent molar ☐
Other ☐
Definition:
______________________</td><td rowspan="4">Clinical examination ☐
Dental casts ☐
Radiographs ☐
Type:
______________________</td></tr>
<tr><td>alveolar ridge ☐</td><td>Cementoenemal junction ☐
Other ☐
Definition:
______________________</td></tr>
<tr><td>alveolar process ☐</td><td>Superior reference:
____
Inferiorer Rerference:
________________________</td></tr>
<tr><td>skeletal growth pattern ☐</td><td>Landmarks:</td></tr>
</table>
