# Supplementary material for: Qualitative and quantitative assessment of infraoccluded deciduous teeth: a systematic review
Source: Head Face Med. 2024 Oct 30;20:65. doi: 10.1186/s13005-024-00469-3 (PMC11524026; doi:10.1186/s13005-024-00469-3)
Supplement: Supplementary file 4 — Supplementary Material 4 [file 13005_2024_469_MOESM4_ESM.pdf]

Additional file 4 Characteristics of excluded studies

Study	Reason for exclusion
Adamska et al. 2024	Literature review (diagnostic and treatment of infraoccluded deciduous teeth)
Al-Abdallah et al., 2015	Investigation of dental anomalies, no qualification or quantification of infraoccluded deciduous teeth
Andlaw, 1974	Literature review (prevalence, aetiology, treatment of infraoccluded deciduous teeth)
Andlaw, 1977	Prevalence study, no qualification or quantification of infraoccluded deciduous teeth
Aranha et al., 2004	Prevalence study, no qualification or quantification of infraoccluded deciduous teeth
Arhakis, Boutiou, 2016	Literature review (etiology, diagnosis, consequences, treatment of deciduous ankylosis)
Ashqar et al., 2019	Literature review (etiology, diagnosis, consequences, treatment of deciduous ankylosis)
Baccetti, 1998	Investigation of dental anomalies, no qualification or quantification of infraoccluded deciduous teeth
Baccetti, 1998	Investigation of dental anomalies, no qualification or quantification of infraoccluded deciduous teeth
Becker, Karnei-R'em, Steigman, 1992	Investigation of the dental arch length and midline
Bergström, 1977	Investigation of dental anomalies, infraoccluded deciduous teeth not included
Bertl et al., 2012	Detection of ankylosis of deciduous teeth by resonance frequency analysis, no qualification or quantification of infraoccluded deciduous teeth
Biavati et al., 2011	Duplicate
Bilinksa, Zadurska, Czochrowska, 2023	Prevalence study, no qualification or quantification of infraoccluded deciduous teeth
Bjerklin, Kurol, Valentin, 1992	Investigation of dental anomalies, no qualification or quantification of infraoccluded deciduous teeth
Bjerklin, 2019	Treatment modalities, no qualification or quantification of infraoccluded deciduous teeth
Bonin, 1976	Case report
Brabant, 1967	Investigation of dental anomalies, no qualification or quantification of infraoccluded deciduous teeth
Brown, 1964	Case report
Caliskan et al., 2021	Pilot study on artificial intelligence for the detection of infraoccluded deciduous teeth, no qualification or quantification of infraoccluded deciduous teeth
Choi, Lee, Song, 2017	Investigation of dental anomalies, no qualification or quantification of infraoccluded deciduous teeth
Darling, Levers, 1975	Investigation of the pattern of eruption of deciduous teeth, premolars and permanent molars, no infraoccluded deciduous teeth included
de Moura, 2015	Case report
Dechaume, 1948	Prevalence study, no qualification or quantification of infraoccluded deciduous teeth
Dixon, 1974	number of subjects with infraoccluded deciduous teeth n=6
Dorward, Sprinz, 1947	Case report
Dos Santo et al., 2022	Systematic review (survival rate of infraoccluded deciduous teeth with aplasia of the successor)

Douglass, Tinanoff, 1991	Literature review (etiology, prevalence, consequences of infraoccluded deciduous teeth)
Dürwald, Köbel, Dannhauer, 2020	Literature review (etiology, characteristics of eruption disorders)
Ekim, Hatibovic-Kofman, 2001	Literature review (treatment of infraoccluded deciduous teeth)
Faramarzi et al., 2019	Prevalence study, no qualification or quantification of infraoccluded deciduous teeth
Feaver, 1949	Investigation of retained deciduous teeth, no infraoccluded deciduous teeth included
Fritzsche, 1970	Case series
Gabka, Kaspar, 1972	Prevalence study, no qualification or quantification of infraoccluded deciduous teeth
Garib, Peck, Gomes, 2009	Investigation of dental anomalies, no qualification or quantification of infraoccluded deciduous teeth
Grivu et al., 1967	Assessment of the eruption of permanent teeth, no infraoccluded deciduous teeth included
Haase, 1970	Case report
Halterman, 2013	Comment
Hekmatfar et al., 2018	Investigation of dental anomalies, no infraoccluded deciduous teeth included
Henderson, 1979	Histological examination of infraoccluded deciduous teeth
Henklein et al., 2023	Investigation of retained deciduous teeth, no infraoccluded deciduous teeth included
Hoffmeister, 1983	Case series
Hotz, 1980	Preservation of deciduous teeth, no infraoccluded deciduous teeth included
Hua et al., 2019	Literature review (treatment of infraoccluded deciduous teeth)
Hudson et al., 2007	Literature review (etiology, prevalence, diagnosis, treatment of infraoccluded deciduous teeth)
Hvaring, Birkeland, 2020	Investigation of the survival rate of infraoccluded deciduous teeth in hypodontia, no qualification or quantification of infraoccluded deciduous teeth
Ith-Hansen, Kjær, 2000	Investigation of the survival rate of infraoccluded deciduous teeth with aplasia of the successor, no qualification or quantification of infraoccluded deciduous teeth
Kennedy, 1951	Case report
Kennedy, 2009	Literature review (treatment of infraoccluded deciduous teeth)
Kravitz, 2019	Comment
Kotsomitis, Freer, 1997	Investigation of dental anomalies, no qualification or quantification of infraoccluded deciduous teeth
Krawiak, 1987	Prevalence study, no qualification or quantification of infraoccluded deciduous teeth
Küchler et al., 2008	Investigation of dental anomalies, no qualification or quantification of infraoccluded deciduous teeth
Kurol, 1981	Prevalence study, no qualification or quantification of infraoccluded deciduous teeth
Kurol, 2002	Literature review (treatment of infraoccluded deciduous teeth)
Kurol, 2006	Literature review (treatment of infraoccluded deciduous teeth)

Kurol, Magnusson, 1984	Histological examination of infraoccluded deciduous teeth
Lai, Seow, 1989	Investigation of dental anomalies, no qualification or quantification of infraoccluded deciduous teeth
Lamb, Reed, 1968	Investigation of the dental arch length
Loudon, 1987	Treatment of infraoccluded deciduous teeth, no infraoccluded deciduous teeth included
McGeown, O'Connell, 2014	Literature review (treatment of infraoccluded deciduous teeth)
Mew, 2012	Comment
Miethke, 1972	Case report
Mohammed, Hashim, Al-Essa, 2018	Literature review (infraoccluded deciduous teeth with aplasia of the successor)
Mueller, 1975	Case series
Mueller et al., 1983	Prevalence study, no qualification or quantification of infraoccluded deciduous teeth
Mufson, Bassiouny, Torreti, 1984	Case series
Murtaugh, 2013	Comment
Noble, Karaiskos, Wiltshire, 2007	Literature review (treatment of infraoccluded deciduous teeth)
Nordquist, Lennartsson, Paulander, 2005	Prevalence study, no qualification or quantification of infraoccluded deciduous teeth
Park, 1979	Case report
Patano et al., 2023	Systematic review (treatment of infraoccluded deciduous teeth)
Pönitz, 1973	Investigation of retained deciduous teeth, no infraoccluded deciduous teeth included
Ristaniemi et al., 2023	Investigation of dental anomalies, no qualification or quantification of infraoccluded deciduous teeth
Rubra, 1972	Comment
Rule, Zacherl, Pfefferle, 1972	Investigation of dental anomalies, no qualification or quantification of infraoccluded deciduous teeth
Rune, Sarnäs, 1984	Investigation of the survival rate of infraoccluded deciduous teeth, no qualification or quantification of infraoccluded deciduous teeth
Sabri, 2008	Literature review (treatment of infraoccldued deciduous teeth)
Savoldi et al., 2021	Systematic review (treatment of infraoccluded deciduous teeth)
Sahyoun, 1968	Case report
Seelinger, 1975	Case report
Sheller, Omnell, 1991	Clinical study on the use of ankylosed deciduous teeth for maxillary protraction, no qualification or quantification of infraoccluded deciduous teeth
Silling, Keller, Feingold, 1979	Case report
Silva et al., 2014	Duplicate
Souza-Silva et al., 2018	Investigation of dental anomalies, no qualification or quantification of infraoccluded deciduous teeth
Stanton, Sweet, West, 1952	Case report
Steigman, Koyoumdjisky-Kaye, Matrai, 1973	Prevalence study, no qualification or quantification of infraoccluded deciduous teeth
Steigman, Koyoumdjisky-Kaye, Matrai, 1973	Prevalence study, no qualification or quantification of infraoccluded deciduous teeth

Steigman, Koyoumdjisky-Kaye, Matrai, 1974	Investigation of root resorption and the successor of infraoccluded deciduous teeth, no qualification or quantification of infraoccluded deciduous teeth
Teague, Barton, Parry, 1999	Literature review (etiology, diagnosis, consequences of infraoccluded deciduous teeth)
Teague, Barton, Parry, 1999	Literature review (treatment of infraoccluded deciduous teeth)
Tieu et al., 2013	Comment
Tieu et al., 2013	Systematic Review (treatment of infraoccluded deciduous teeth)
Tong et al., 2020	RNA sequencing of infraoccluded deciduous teeth, no qualification or quantification of infraoccluded deciduous teeth
Torlińska-Walkowiak, 2022	Literature review (prevalence, treatment of infraoccluded deciduous teeth)
Tunis et al, 2021	Investigation of dental anomalies, no qualification or quantification of infraoccluded deciduous teeth
Via, 1964	Prevalence study, no qualification or quantification of infraoccluded deciduous teeth
Vorhies, Gergory, McDonald, 1952	Literature review (etiology, diagnosis, treatment of infraoccluded deciduous teeth)
Walshaw et al., 2020	Investigation of dental anomalies, no qualification or quantification of infraoccluded deciduous teeth
Winter, Gelbier, Goodman, 1997	Case series
Wylleman et al., 2020	Examination of the alveolar bone level at deciduous molars, no infraocclduded deciduous teeth included
Yavuz et al., 2023	Investigation of dental anomalies, no qualification or quantification of infraoccluded deciduous teeth

Additional file 2 Reference list of excluded articles

1. Adamska P, Sobczak-Zagalska H, Stasiak M, Adamski Ł, Pylińska-Dąbrowska D, Barkowska S, et al. Infraocclusion in the Primary and Permanent Dentition-A Narrative Review. Medicina (Kaunas, Lithuania). 2024;60(3).

2. Al-Abdallah M, AlHadidi A, Hammad M, Al-Ahmad H, Saleh R. Prevalence and distribution of dental anomalies: a comparison between maxillary and mandibular tooth agenesis. American Journal of Orthodontics and Dentofacial Orthopedics. 2015;148(5):793-8.

3. Andlaw RJ. Submerged deciduous molars. A review, with special reference to the rationale of treatment. Journal of the International Association of Dentistry for Children. 1974;5(2):59-66.

4. Andlaw RJ. Submerged deciduous molars: a prevalence survey in Somerset. Journal of the International Association of Dentistry for Children. 1977;8(2):42-5.

5. Aranha AM, Duque C, Silva JY, Carrara CF, Costa B, Gomide MR. Tooth ankylosis in deciduous teeth of children with cleft lip and/or palate. Braz Oral Res. 2004;18(4):329-32.

6. Arhakis A, Boutiou E. Etiology, Diagnosis, Consequences and Treatment of Infraoccluded Primary Molars. Open Dent J. 2016;10:714-9.

7. Ashqar SJ, Alim AF, Emam LS, Andijani FS, Karsan DA, Khayyat AA, et al. INFRA-OCCLUDED PRIMARY MOLARS MANAGEMENT AND BENEFITS OF AN EARLY DIAGNOSIS. Indo American Journal of Pharmaceutical Sciences. 2019;6(1):2782-8.

8. Baccetti T. A controlled study of associated dental anomalies. Angle Orthodontist. 1998;68(3):267-74.

9. Baccetti T. A clinical and statistical study of etiologic aspects related to associated tooth anomalies in number, size, and position. Minerva Stomatologica. 1998;47(12):655-63.

10. Becker A, Karnei-R'em R M, Steigman S. The effects of infraocclusion: Part 3. Dental arch length and the midline. American Journal of Orthodontics and Dentofacial Orthopedics. 1992;102(5):427-33.

11. Bergstrom K. An orthopantomographic study of hypodontia, supernumeraries and other anomalies in school children between the ages of 8-9 years. An epidemiological study. Swedish Dental Journal. 1977;1(4):145-57.

12. Bertl MH, Weinberger T, Schwarz K, Gruber R, Crismani AG. Resonance frequency analysis: a new diagnostic tool for dental ankylosis. European Journal of Oral Sciences. 2012;120(3):255-8.

13. Biavati AS, Signori A, Castaldo A, Matarese G, Migliorati M. Incidence and distribution of deciduous molar ankylosis, a longitudinal study. European journal of paediatric dentistry : official journal of European Academy of Paediatric Dentistry. 2011;12(3):175-8.

14. Bilinska M, Zadurska M, Czochrowska E. Status of retained deciduous second molars in subjects with agenesis of second premolars in relation to age. European Journal of Paediatric Dentistry. 2023;24(4):262 - 6.

15. Bjerklin K. Orthodontic management of agenesis of mandibular second premolars. Apos Trends in Orthodontics. 2019;9(4):206-10.

16. Bjerklin K, Kurol J, Valentin J. Ectopic eruption of maxillary first permanent molars and association with other tooth and developmental disturbances. European Journal of Orthodontics. 1992;14(5):369-75.

17. Bonin M. SIMPLIFIED AND RAPID TREATMENT OF ANKYLOSED PRIMARY MOLARS WITH AN AMALGAM AND COMPOSITE RESIN. Journal of Dentistry for Children. 1976;43(3):23-6.

18. Brabant H. Comparison of the characteristics and anomalies of the deciduous and the permanent dentition. Journal of Dental Research. 1967;46(5):897-902.

19. Brown JW. SUBMERGED AND RESTORED SECOND DECIDUOUS MOLAR. Journal of the American Dental Association. 1964;68:151.

20. Caliskan S, Tuloglu N, Celik O, Ozdemir C, Kizilaslan S, Bayrak S. A pilot study of a deep learning approach to submerged primary tooth classification and detection. International Journal of Computerized Dentistry. 2021;24(1):1-9.

21. Choi SJ, Lee JW, Song JH. Dental anomaly patterns associated with tooth agenesis. Acta Odontologica Scandinavica. 2017;75(3):161-5.

22. Darling AI, Levers BG. The pattern of eruption of some human teeth. Archives of Oral Biology. 1975;20(2):89-96.

23. de Moura MS, Pontes AS, Brito MH, de Deus Moura L, de Deus Moura de Lima M, de Melo Simplício AH. Restorative management of severely ankylosed primary molars. J Dent Child (Chic). 2015;82(1):41-6.

24. Dechaume M, Cauhepe J. Retention of deciduous molars. Dental Record. 1948;68(7):173-5.

25. Dixon DA. Ethnic variations in the incidence of submerging deciduous molars correlated with vertical dysplasias of the human face and dentition. Transactions of the European Orthodontic Society. 1974:199-205.

26. Dorward JG, Sprinz R. Retention of deciduous molar between first and second lower premolars. British Dental Journal. 1947;83(10):216.

27. Dos Santos CCO, Melo DL, da Silva PP, Normando D. What is the survival rate of deciduous molars in cases with agenesis of premolar successors? A systematic review. Angle Orthodontist. 2022;92(1):110-7.

28. Douglass J, Tinanoff N. The etiology, prevalence, and sequelae of infraclusion of primary molars. ASDC Journal of Dentistry for Children. 1991;58(6):481-3.

29. Dürwald J, Köbel C, Dannhauer KH. Tooth Eruption Disorders - Causes and Symptoms. Informationen Aus Orthodontie Und Kieferorthopaedie. 2020;52(1):35-55.

30. Ekim SL, Hatibovic-Kofman S. A treatment decision-making model for infraoccluded primary molars. International Journal of Paediatric Dentistry. 2001;11(5):340-6.

31. Faramarzi H, Damankesh Z, Mousavi-Roknabadi RS, Keshavarz S. Retained primary tooth in unusual ages in southern Iran: A population-based study. Journal of Oral Health and Oral Epidemiology. 2019;8(2):89-96.

32. Feaver RE. Retained deciduous teeth. British Dental Journal. 1949;86(6):153.

33. Fritzsche H. [Inclusion of deciduous molars. Growth inhibition or recession]. Deutsche Stomatologie. 1970;20(10):771-6.

34. Gabka J, Kaspar E. [Impacted and ectopic teeth. A 10-year evaluation at the Rudolf Virchow Hospital--without consideration of wisdom teeth]. Deutsche Stomatologie. 1972;22(12):925-35.

35. Garib DG, Peck S, Gomes SC. Increased occurrence of dental anomalies associated with second-premolar agenesis. Angle Orthodontist. 2009;79(3):436-41.

36. Grivu O, Theiss E, Ghibu-Petcu C, Mecher E, Patrut G. [Contribution to the study on the changing of teeth]. Deutsche Zahnarztliche Zeitschrift. 1967;22(9):1120-8.

37. Haase G. [Retention of deciduous teeth]. Deutsche Stomatologie. 1971;21(1):45-7.

38. Halterman C. Ankylosed primary molars. Journal of the American Dental Association. 2013;144(9):978.

39. Hekmatfar S, Bagheri A, Jafari K, Zarei S, Heidarzadeh Z. Incidence of dental developmental anomalies in permanent dentition among Ardabil population, Iran, in 2015-2016. Journal of Oral Health and Oral Epidemiology. 2018;7(2):64-8.

40. Henderson HZ. Ankylosis of primary molars: a clinical, radiographic, and histologic study. ASDC Journal of Dentistry for Children. 1979;46(2):117-22.

41. Henklein SD, Küchler EC, Proff P, Lepri CP, Baratto-Filho F, Mattos NHR, et al. Prävalenz und lokale Ursachen für die Retention von Milchzähnen und der damit verbundenen Verzögerung des Durchbruchs bleibender Zähne. Journal of orofacial orthopedics = Fortschritte der Kieferorthopadie : Organ/official journal Deutsche Gesellschaft fur Kieferorthopadie. 2023.

42. Hoffmeister H. [Tongue pressure or inherited susceptibility as a primary cause of infraclusion or impaction of deciduous molars?]. Fortschritte der Kieferorthopädie. 1983;44(4):316-25.

43. Hotz PR. [Retention of the deciduous molars]. SSO: Schweizerische Monatsschrift für Zahnheilkunde. 1980;90 Spec No:803-13.

44. Hua L, Thomas M, Bhatia S, Bowkett A, Merrett S. To extract or not to extract? Management of infraoccluded second primary molars without successors. British Dental Journal. 2019;227(2):93-8.

45. Hudson AP, Harris AM, Morkel JA, Amra I. Infraocclusion of primary molars: a review of the literature. SADJ. 2007;62(3):114, 6, 8-22.

46. Hvaring CL, Birkeland K. The long-term fate of persisting deciduous molars and canines in 42 patients with severe hypodontia: a 12-year follow-up. European Journal of Orthodontics. 2020;42(6):581-6.

47. Ith-Hansen K, Kjaer I. Persistence of deciduous molars in subjects with agenesis of the second premolars. European Journal of Orthodontics. 2000;22(3):239-43.

48. Kennedy DB. Treatment strategies for ankylosed primary molars. European Archives of Paediatric Dentistry. 2009;10(4):201-10.

49. Kennedy DJ. Multiple impactions of permanent bicuspids and deciduous molars. Journal of the American Dental Association. 1951;43(2):209-11.

50. Kotsomitis N, Freer TJ. Inherited dental anomalies and abnormalities. Journal of Dentistry for Children. 1997;64(6):405-+.

51. Krakowiak FJ. Ankylosed primary molars. ASDC Journal of Dentistry for Children. 1978;45(4):288-92.

52. Kravitz ND. Composite Build-Up of Infraoccluded Second Deciduous Molars. Journal of Clinical Orthodontics. 2019;53(5):290.

53. Küchler EC, Risso PA, Costa Mde C, Modesto A, Vieira AR. Studies of dental anomalies in a large group of school children. Archives of Oral Biology. 2008;53(10):941-6.

54. Kurol J. Infraocclusion of primary molars: an epidemiologic and familial study. Community Dentistry and Oral Epidemiology. 1981;9(2):94-102.

55. Kurol J. Early treatment of tooth-eruption disturbances. American Journal of Orthodontics and Dentofacial Orthopedics. 2002;121(6):588-91.

56. Kurol J. Impacted and ankylosed teeth: why, when, and how to intervene. American Journal of Orthodontics and Dentofacial Orthopedics. 2006;129(4 Suppl):S86-90.

57. Kurol J, Magnusson BC. Infraocclusion of primary molars: a histologic study. Scandinavian Journal of Dental Research. 1984;92(6):564-76.

58. Lai PY, Seow WK. A controlled study of the association of various dental anomalies with hypodontia of permanent teeth. Pediatric Dentistry. 1989;11(4):291-6.

59. Lamb KA, Reed MW. Measurement of space loss resulting from tooth ankylosis. ASDC Journal of Dentistry for Children. 1968;35(6):483-6.

60. Loudon ME. Vertical dimension--primary molar buildup. Functional Orthodontist. 1987;4(3):38-9.

61. McGeown M, O'Connell A. Management of primary molar infraocclusion in general practice. Journal of the Irish Dental Association. 2014;60(4):192-8.

62. Mew J. Infraoccluded deciduous molars. American Journal of Orthodontics and Dentofacial Orthopedics. 2012;141(4):395-6.

63. Miethke RR. [Retention of a deciduous tooth]. Deutsche Zahnarztliche Zeitschrift. 1972;27(8):693-5.

64. Mohammed DR, Hashim RS, Al-Essa HS. Retention of Primary Second Molars without a Permanent Successor: A Review Article. International Journal of Medical Research & Health Sciences. 2018;7(7):80-9.

65. Mueller CT, Gellin ME, Kaplan AL, Bohannan HM. Prevalence of ankylosis of primary molars in different regions of the United States. ASDC Journal of Dentistry for Children. 1983;50(3):213-8.

66. Mueller GH. Retention of deciduous teeth. II. Fortschritte der Kieferorthopädie. 1975;36(2):127-34.

67. Mufson RA, Bassiouny MA, Torreti EF. ALTERNATE RESTORATIVE TECHNIQUES FOR MANAGEMENT OF OVER-RETAINED ANKYLOSED PRIMARY TEETH. Journal of the American Dental Association. 1984;108(6):983-5.

68. Murtaugh J. Submerged primary molars. Journal of the American Dental Association. 2013;144(9):978.

69. Noble J, Karaiskos N, Wiltshire WA. Diagnosis and management of the infraerupted primary molar. British Dental Journal. 2007;203(11):632-4.

70. Norquist I, Lennartsson B, Paulander J. Primary teeth in adults - A pilot study. Swedish Dental Journal. 2005;29(1):27-34.

71. Park JK. SUBMERGED IMPACTED PRIMARY MOLAR. Oral Surgery Oral Medicine Oral Pathology Oral Radiology and Endodontics. 1979;48(4):383-.

72. Patano A, Inchingolo AM, Laudadio C, Azzollini D, Marinelli G, Ceci S, et al. Therapeutic Strategies of Primary Molar Infraocclusion: A Systematic Review. Children (Basel). 2023;10(3).

73. Pönitz W. [Multiple tooth retention]. Deutsche Stomatologie. 1973;23(5):347-50.

74. Ristaniemi J, Kujasalo K, Rytkönen E, Melaluoto E, Iivari J, Pesonen P, et al. Features of Dental Anomaly Patterns in Finnish children as seen in panoramic radiographs at the late mixed stage. Acta Odontologica Scandinavica. 2023;81(8):609-14.

75. Rubra CH. RETENTION OF PRIMARY TEETH. British Dental Journal. 1972;132(3):91-&.

76. Rule JT, Zacherl WA, Pfefferle AM. The relationship between ankylosed primary molars and multiple enamel defects. ASDC Journal of Dentistry for Children. 1972;39(1):29-35.

77. Rune B, Sarnäs KV. Root resorption and submergence in retained deciduous second molars. A mixed-longitudinal study of 77 children with developmental absence of second premolars. European Journal of Orthodontics. 1984;6(2):123-31.

78. Sabri R. Management of over-retained mandibular deciduous second molars with and without permanent successors. World Journal of Orthodontics. 2008;9(3):209-20.

79. Sahyoun I. 'Descending' deciduous molar and impacted second molar. Oral Surgery, Oral Medicine, Oral Pathology. 1968;26(2):191-2.

80. Savoldi F, Dalessandri D, Gardoni A, Dianiskova S, Bonetti S, Visconti L. Treatment of ankylosed deciduous molars with or without permanent successors in children and adolescents: a systematic review. Minerva Dent Oral Sci. 2021;70(6):276-85.

81. Seelinger JE. Embedded primary maxillary second molar tooth. Oral Surgery, Oral Medicine, Oral Pathology. 1975;40(6):818-9.

82. Sheller B, Omnell L. Therapeutic ankylosis of primary teeth. Journal of Clinical Orthodontics. 1991;25(8):499-502.

83. Silling G, Keller JG, Feingold M. Retained primary teeth: their effect on developing occlusions. ASDC Journal of Dentistry for Children. 1979;46(4):296-9.

84. Silva CC, Edo MM, Llorente MSA, Leache EB. Primary molar infraocclusion: frequency, magnitude, root resorption and premolar agenesis in a Spanish sample. European Journal of Paediatric Dentistry. 2014;15(3):258-64.

85. Souza-Silva BN, Vieira WA, Bernardino Í M, Batista MJ, Bittencourt MAV, Paranhos LR. Non-syndromic tooth agenesis patterns and their association with other dental anomalies: A retrospective study. Archives of Oral Biology. 2018;96:26-32.

86. Stanton EM, Sweet CA, West FT. Submerged deciduous molars. Journal of the American Dental Association. 1952;46(4):443-5.

87. Steigman S, Koyoumdjisky Kaye E, Matrai Y. Submerged deciduous molars and congenital absence of premolars. Journal of Dental Research. 1973;52(4):842.

88. Steigman S, Koyoumdjisky-Kaye E, Matrai Y. Submerged deciduous molars in preschool children: an epidemiologic survey. Journal of Dental Research. 1973;52(2):322-6.

89. Steigman S, Koyoumdjisky-Kaye E, Matrai Y. Relationship of submerged deciduous molars to root resorption and development of permanent successors. Journal of Dental Research. 1974;53(1):88-93.

90. Teague AM, Barton P, Parry WJ. Management of the submerged deciduous tooth: I. Aetiology, diagnosis and potential consequences. Dental Update. 1999;26(7):292-6.

91. Teague AM, Barton P, Parry WJ. Management of the submerged deciduous tooth: 2. Treatment. Dental Update. 1999;26(8):350-2.

92. Tieu LD, Walker SL, Major MP, Flores-Mir C. Management of ankylosed primary molars with premolar successors: a systematic review. Journal of the American Dental Association. 2013;144(6):602-11.

93. Tieu LD, Walker SL, Major MP, Flores-Mir C. SUBMERGED PRIMARY MOLARS response. Journal of the American Dental Association. 2013;144(9):978-9.

94. Tong AN, Chow YL, Xu KT, Hardiman R, Schneider P, Tan SS. Transcriptome analysis of ankylosed primary molars with infraocclusion. International journal of oral science. 2020;12(1).

95. Torlińska-Walkowiak N, Tuczyńska M, Kucharska K, Wyzga S, Singh N, Łukomska-Pochylska M, et al. Infraocclusion – an anomaly in primary dentition. Pediatria i Medycyna Rodzinna. 2022;18(4):341-4.

96. Tunis TS, Sarne O, Hershkovitz I, Finkelstein T, Pavlidi AM, Shapira Y, et al. Dental anomalies' characteristics. Diagnostics. 2021;11(7).

97. Via WF, Jr. SUBMERGED DECIDUOUS MOLARS: FAMILIAL TENDENCIES. Journal of the American Dental Association. 1964;69:127-9.

98. Vorhies JM, Gregory GT, Mc DR. Ankylosed deciduous molars. Journal of the American Dental Association. 1952;44(1):68-72.

99. Walshaw EG, Noble F, Conville R, Anne Lawson J, Hasmun N, Rodd H. Molar incisor hypomineralisation and dental anomalies: A random or real association? International Journal of Paediatric Dentistry. 2020;30(3):342-8.

100. Winter GB, Gelbier MJ, Goodman JR. Severe Infra-occlusion and failed eruption of deciduous molars associated with eruptive and developmental disturbances in the permanent dentition: a report of 28 selected cases. British Journal of Orthodontics. 1997;24(2):149-57.

101. Wylleman A, Van der Veken D, Teughels W, Quirynen M, Laleman I. Alveolar bone level at deciduous molars in Flemish children: A retrospective, radiographic study. Journal of Clinical Periodontology. 2020;47(6):660-7.

102. Yavuz BS, Sezer B, Kaya R, Tugcu N, Kargül B. Is there an association between molar incisor hypomineralization and developmental dental anomalies? A case-control study. BMC Oral Health. 2023;23(1).
